# Patient Parameters Associated With a Positive Blood Culture Using NeuroShell: A Retrospective Chart Review

**DOI:** 10.7759/cureus.28635

**Published:** 2022-08-31

**Authors:** Richard Giovane, Robert A Sheppard

**Affiliations:** 1 Family Medicine, University of Alabama at Birmingham, Tuscaloosa, USA; 2 Internal Medicine, University of Alabama at Birmingham, Tuscaloosa, USA

**Keywords:** machine learning healthcare data, positive blood culture, severe sepsis, deep neural network, persistent bacteremia

## Abstract

Bacteremia is a common and life-threatening condition. It has an incidence of 140 to 160 per 100,000 person-years in the United States. Since bacteremia has many presentations, it can be challenging to diagnose. Subsequently there are very few guidelines on when to order a blood culture in an emergency setting. Neural networks are a means of machine learning and are presently being used in medicine to aid in decision making. With the use of machine learning, 22 variables that have been associated with infection and bacteremia were used to build a neural network to determine which variables associated with bacteremia are most associated with a positive blood culture.

## Introduction

Knowing when it is appropriate to order a blood culture for a patient in an emergency setting can be challenging. Bacteremia is common in the emergency room, with an incidence of 140 to 160 per 100,000 person-years in the United States [1]. Due to its high incidence, it is important in determining a means to accurately diagnose bacteremia. Diagnosing bacteremia is challenging in that bacteremia has a wide spectrum of presentations such as fever, tachycardia, hypotension, tachypnea, altered mental status and oliguria [2]. There are however few guidelines on when to order a blood culture [1,3,4]. A decision rule was created by Shapiro et al. in 2008 which defined major and minor criteria; “major criteria” are defined as: temperature > 39.5°C (103.0°F), indwelling vascular catheter, or clinical suspicion of endocarditis [3]. “Minor criteria” are: temperature 38.3-39.4°C (101-102.9°F), age > 65 years, chills, vomiting, hypotension (systolic blood pressure < 90 mm Hg), neutrophil % > 80, white blood cell count > 18k, bands > 5%, platelets < 150 k, and creatinine > 2.0. A blood culture is indicated by this rule if at least one major criterion or two minor criteria are present [1,3]. This decision rule is useful, however it does have limitations [3]. There was also a scoping review done by Fabre et al. that created a pretest probability of syndromes that grouped them into low, moderate or high pretest probability of bacteremia [4]. Although useful, it is only a scoping review and its practicality has not been tested. 

With few clinical guidelines on when to order a blood culture, it can lead to over-ordering blood cultures, an increased false positive rate and initiation of antibiotics in patients that do not require them [5]. Moreover it can increase costs to hospitals due to over-testing [5,6-8]. 

The use of deep learning and neural networks are being utilized in medicine to help solve medical problems by giving input in medical decisions and reducing costs. Deep learning is a hierarchical method of learning in that data is supplied to continuous layers which have output from previous layers so that each subsequent layer learns from the previous layer [9]. Neural networks make up the core of deep learning as it mimics animal neuron pathways in that one neuron is stimulated by output of a previous neuron. Through continuous layers in a neural network, the inputted data can traverse several layers within the neural network to optimize output [9]. The neural network will continuously learn more as more data enters the neural network. Neural networks are already being implemented in medicine to aid in clinical decision making [10]. As an example, the neural network ICU Intervene obtains patient parameters from vital signs and laboratory values which then generates predictions on if an ICU patient will require intubation within six hours [10]. 

The aim of this study was to utilize a neural network to clinically assess patient data. The neural network, through deep learning, would then determine which variables, associated with bacteremia are most highly associated with a positive blood culture.

## Materials and methods

This study was a retrospective chart review. Charts were reviewed of patients that met inclusion criteria which was any patient who was 18 years or older and had a positive blood culture that was not documented as a contaminate. They also needed to be admitted at DCH Regional Medical Center in Tuscaloosa, Alabama, from January 2016 to January 2018. Five hundred ten charts were reviewed and data were extracted based on 22 specific variables that have been associated with infection or bacteremia [1,2,6-8]. The variables that were included were 1) Patient having cancer and receiving active chemotherapy or radiation therapy or the patient has an immunocompromised state such as AIDS, autoimmune disorders, acquired immunodeficiency, organ transplant or on immunosuppression therapy; 2) Fever (temp >100.4℉); 3) quick sequential organ failure assessment (qSOFA) score (respiratory rate ≥ 22 breaths/min, altered mental status [Glascow Coma Scale <15], or systolic blood pressure [SBP] ≤ 100 mm); 4) Recent hospitalizations within the past 30 days; 5) White blood cell count <4000 or >12000 (measured in x109 /L); 6) Age >65; 7) Documented erythema on physical exam; 8) New onset cough within the past seven days; 9) Documented infected wound; 10) Altered mental status (Glascow Coma Scale <15); 11) Hypotension (SBP ≤100mmHg); 12) Documented sick appearing patient; 13) New onset shortness of breath; 14) New onset abdominal pain; 15) Respiratory failure/tachypnea (defined as partial pressure of oxygen [Pa02] ≤60mmgHg or PaCO2 ≥50mmHg or RR≥ 22); 16) New onset dysuria; 17) Lactic acid level (If not obtained, a value of normal: 0.7 was assigned); 18) History of Diabetes Mellitus type 2; 19) Documented abscess on physical exam; 20) Patient in the ICU/sent to ICU after initial triage; 21) Tachycardia (HR ≥100 BPM); 22) Creatinine level, measured in mg/dL. All data was kept in an encrypted spreadsheet. Any non-numerical value was given a value of 0 for “No” or 1 for Yes”. Data was then transferred to NeuroShell Classifier© (Wardsystems, Frederick, MD, USA). This software is used to create a neural network for learning and to solve decision-making problems and creates a neural network that learns from observational data. The neural network, which was named BL@ckLotus for this study, created a neural network based on the input data to learn important associations with having a positive blood culture. The primary end point of this study was to create a neural network to clinically assess patient data, utilize the data through deep learning and then determine the most important variables that are associated with a positive blood culture.

## Results

**Figure 1:**
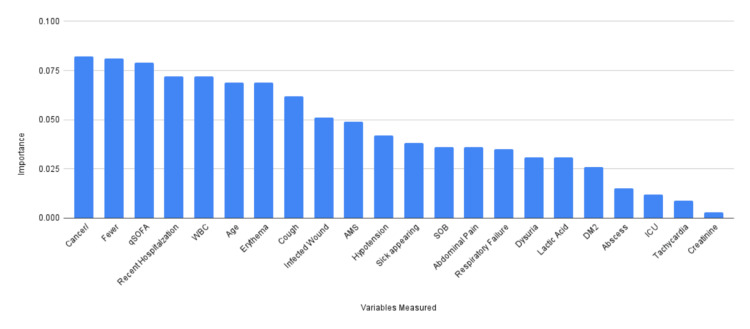
Twenty-two variables measured by Bl@ckLotus and the importance of each variable as determined by NeuroShell Cancer/ IC- Cancer/Immunocompromised Recent Hosp- Recent Hospitalization <30 days WBC- White blood cell count AMS- Altered Mental Status SOB- Shortness of breath DM2- Diabetes mellitus type 2 ICU- ICU stay

After all 510 patient variables were put into a spreadsheet, the data was then utilized by NeuroShell to create a neural network. From this inputted data, NeuroShell determined the variables with the highest association of having a positive blood culture (Table 1, Figure 1).

**Table 1:** List of variables in order of importance as determined by NeuroShell. qSOFA: quick sequential organ failure assessment

Variable	Importance
Cancer/ Immunocompromised	0.082
Fever	0.081
qSOFA	0.079
Recent Hospitalzation <30d	0.072
White blood cell count	0.072
Age	0.069
Erythema	0.069
Cough	0.062
Infected wound	0.051
Altered mental status	0.049
Hypotension	0.042
Sick appearing	0.038
Shortness of breath	0.036
Abdominal pain	0.036
Respiratory failure	0.035
Dysuria	0.031
Lactic acid	0.031
Type 2 diabetes mellitus	0.026
Abscess	0.015
In the ICU	0.012
Tachycardia	0.09
Creatinine	0.03

## Discussion

The decision to order a blood culture can be difficult as there is limited guidance in the form of guidelines as well as decision rules [1-3]. As many conditions, such as sepsis, require the physician to order a blood culture in a timely manner prior to antibiotic use, it is imperative that the decision to order a blood culture be medically sound and in a patient’s best interest [7]. Moreover, testing can accrue costs to a hospital, so having a strategy of when it is best to order a blood culture would help hospitals financially [5,6]. 

The results found in this study are encouraging regarding which variables have the highest association with a positive blood culture. Although limited in this study, NeuroShell did make predictions on which variables had the highest association with a positive blood culture. The highest associated variable of a positive blood culture was the patient having a history of cancer or on chemotherapy or being immunocompromised. Having a compromised immune system leads to the patient being susceptible to infections and potentially bacteremia [10,11]. 

Fever was the second most important variable. Although there are many non-infectious causes of fever, fever can indicate an infection given the right clinical setting, such as bacteremia [12]. 

A high qSOFA score had the third highest association. This scoring system was developed to assess a non-ICU patient’s mortality from sepsis [13]. This is done at bedside and is scored from 0-3. A patient is given one point if their respiratory rate is ≥22, or if they have altered mental status [Glasgow coma scale <15] or if their systolic blood pressure ≤100 mmHg. Although qSOFA is not diagnostic of sepsis, it can determine their mortality, with a score of 2 or greater having a poor outcome [13,14]. 

A patient who has recently been hospitalized has an increased risk of developing a hospitalized acquired infection (HAI) [15]. The risk of developing an HAI is dependent on age, immune status, antibiotic use, length of stay and comorbidities and the types of infection vary and can include pneumonia, bacteremia, or urinary tract infections [16]. In 2014, the CDC reported that 4% of patients in the hospital had HAI [15,16]. Interestingly, NeuroShell found that a white blood cell count of <4000 or >12000x109 /L had an equal association of a positive blood culture as a recent hospitalization. Leukopenia and leukocytosis are highly associated with infection which would be suspected in bacteremia. 

Both age and presence of erythema on physical exam had an equal association of a positive blood culture. Elderly patients have an increased risk of developing an infection due to a decrease in immune response from a decline in humoral immunity and cytokine production [17,18]. Moreover, elderly patients have an increased rate of chronic conditions such as type 2 diabetes, heart disease and cancer that decrease an immune response [18]. The presence of erythema on a physical exam has many causes however an infectious cause is always worrisome for developing a soft tissue infection which can worsen [19]. 

The other variables listed had a lower association with a positive blood culture than the ones mentioned previously. Although important in this study, there is much variance in their association with bacteremia.

There were many limitations of this study. The first limitation of this study was the power of the study. Only 510 patients were used in this study. Another limitation is the patient demographic used. The state of Alabama has the second highest rate of type 2 diabetes as well as it has the third highest rate of hypertension [20]. Conversely, Alabama is ranked 11th in HIV diagnoses per 100,000 people [20]. As Alabama has a different prevalence of disease, this can skew data as there was not an even distribution of disease studied. It would be welcomed in further studies to sample patients from different states to adjust for certain diseases and have a balanced representation. Another limitation to this study was that it was a retrospective chart review. In that regard, the investigators had to rely strictly on what was documented in the chart. As an example, one variable looked at whether the patient “looked sick”. This was up to the discretion of the physician that documented the patient’s physical exam. There is potential for error as a physician’s definition of “looking sick” can differ. Another limitation was regarding the variable creatinine levels documented. Some patients in this study did have a history of chronic kidney disease, therefore their creatinine levels would be elevated regardless if they had a positive blood culture. Another limitation was that only patients who had a positive blood culture were used. This does drastically limit deep learning of the neural network due to the neural network only learning from a positive outcome: a positive blood culture. Lastly, the aim of this study was to use a neural network to utilize data in the form of a clinical assessment to make predictions on which variables have the highest association with a positive blood culture. The data is very encouraging and congruent with the literature however it does have its limitations. The scope of this study was to create a neural network to learn associations of a positive blood culture however future studies would be welcomed to use this data to create a scoring system or clinical decision rule on when to order a blood culture. 

## Conclusions

The use of neural networks as a clinical assessment tool, can be utilized in understanding which variables are highly associated with a positive blood culture. Although this was a small study, the variables most highly associated with a positive blood culture were immunocompromised state, fever, an elevated qSOFA score, recent hospitalization within 30 days and abnormal white blood cell count. Although these findings were limited, future studies in creating a clinical decision rule utilizing the data from this neural network would be welcomed.
